# Symptoms, the Nature of Fibromyalgia, and *Diagnostic and Statistical Manual 5 (DSM-5)* Defined Mental Illness in Patients with Rheumatoid Arthritis and Fibromyalgia

**DOI:** 10.1371/journal.pone.0088740

**Published:** 2014-02-14

**Authors:** Frederick Wolfe, Brian T. Walitt, Robert S. Katz, Winfried Häuser

**Affiliations:** 1 National Data Bank for Rheumatic Diseases and University of Kansas School of Medicine, Wichita, Kansas, United States of America; 2 Washington Hospital Center, Washington, D.C., United States of America; 3 Rush University Medical Center, Chicago, Illinois, United States of America; 4 Department of Psychosomatic Medicine and Psychotherapy Technische Universität München, Munich, Germany; 5 Department Internal Medicine I, Klinikum Saarbrücken, Saarbrücken, Germany; University of Texas Health Science Center at Houston, United States of America

## Abstract

**Purpose:**

To describe and evaluate somatic symptoms in patients with rheumatoid arthritis (RA) and fibromyalgia, determine the relation between somatization syndromes and fibromyalgia, and evaluate symptom data in light of the Diagnostic and Statistical Manual-5 (DSM-5) criteria for somatic symptom disorder.

**Methods:**

We administered the Patient Health Questionnaire-15 (PHQ-15), a measure of somatic symptom severity to 6,233 persons with fibromyalgia, RA, and osteoarthritis. PHQ-15 scores of 5, 10, and 15 represent low, medium, and high somatic symptom severity cut-points. A likely somatization syndrome was diagnosed when PHQ-15 score was ≥10. The intensity of fibromyalgia diagnostic symptoms was measured by the polysymptomatic distress (PSD) scale.

**Results:**

26.4% of RA patients and 88.9% with fibromyalgia had PHQ-15 scores ≥10 compared with 9.3% in the general population. With each step-wise increase in PHQ-15 category, more abnormal mental and physical health status scores were observed. RA patients satisfying fibromyalgia criteria increased from 1.2% in the PHQ-15 low category to 88.9% in the high category. The sensitivity and specificity of PHQ-15≥10 for fibromyalgia diagnosis was 80.9% and 80.0% (correctly classified = 80.3%) compared with 84.3% and 93.7% (correctly classified = 91.7%) for the PSD scale. 51.4% of fibromyalgia patients and 14.8% with RA had fatigue, sleep or cognitive problems that were severe, continuous, and life-disturbing; and almost all fibromyalgia patients had severe impairments of function and quality of life.

**Conclusions:**

All patients with fibromyalgia will satisfy the DSM-5 “A” criterion for distressing somatic symptoms, and most would seem to satisfy DSM-5 “B” criterion because symptom impact is life-disturbing or associated with substantial impairment of function and quality of life. But the “B” designation requires special knowledge that symptoms are “disproportionate” or “excessive,” something that is uncertain and controversial. The reliability and validity of DSM-5 criteria in this population is likely to be low.

## Introduction

Fibromyalgia is defined by criteria that depend upon the range and severity of symptoms. Because the defining symptom of fibromyalgia is widely distributed pain, fibromyalgia is usually considered a pain disorder, at least in the rheumatology and pain communities. In the American College of Rheumatology (ACR) 1990 fibromyalgia criteria [1], musculoskeletal pain is the only symptom evaluated, while in the updated 2010 criteria [2], [3], only 1 of the 5 criteria items directly concerns musculoskeletal pain. In other disciplines–particularly psychiatry, psychology, psychosomatic medicine and, perhaps, general medicine, fibromyalgia is more often considered to be a symptom or psychosomatic disorder [4], [5].

Symptom disorders–simply the presence of many symptoms–are related to the concept of somatization that organizes and attributes meaning to them. Lipowski’s often cited summary, defined somatization as “… a tendency to experience and communicate somatic distress and symptoms unaccounted for by pathological findings, to attribute them to physical illness, and to seek medical help for them,” adding that “it is usually assumed that this tendency becomes manifest in response to psychosocial stress brought about by life events and situations that are personally stressful to the individual” [4]. In his comprehensive review he warned, however, that “… symptoms are extremely common and do not necessarily indicate that the person experiencing them is under stress or interprets them as suggestive of physical illness and seeks medical help.” Moreover, somatization “… is neither a disorder nor a diagnostic category and does not imply that an individual displaying it must suffer from a psychiatric disorder” [4]. But in practical terms, psychological issues are not to be ignored: “the real divide in defining somatization, wrote Kurt Kroenke, centers on the role of psychologic factors” [5]. *Somatization*–the disease not just the concept was officially enshrined as a psychiatric diagnosis in the American Psychiatry Association’s Disease and Statistical Manual of Mental disorders versions 3 through 4 as one of the somatoform disorders, (DSM-III. DSM-IV, DSM-IVTR), [6], [7], [8] but was abandoned in 2013 in DSM-5 [9] and replaced by “Somatic Symptom Disorder” (DSM SSD) (See Table S1).

Somatization as a disorder faltered when it was recognized that the idea of “unexplained” physical symptoms was not intellectually tenable and it became clear that “medically unexplained symptoms” and “symptoms unaccounted for by pathological findings” implied inauthenticity (“It’s all in your head)” [10]. In addition, ‘somatization’ criteria couldn’t adequately handle symptoms that were not unexplained but which were potentially “exaggerated.” DSM-IV criteria for somatization were so arcane and difficult to satisfy that the diagnosis was almost never made, while DSM-IV “*Undifferentiated Somatoform Disorder*” criteria were too easy to satisfy [10]. The prevalence of undifferentiated somatoform disorder in the general population was 20% [11], 25% in neurology outpatients [12], and 27% in primary care [13]. Still, despite the clear occurrence of patients with somatization and undifferentiated somatoform disorders in the clinic, these DSM-IV illnesses did not gain diagnostic acceptance and were rarely coded [10]. Among researchers there were attempts to rename somatic symptom disorders to somatic symptoms disorder [14], physical symptom disorder [15], functional symptom disorder [16], bodily distress syndrome [17], among others. The idea of somatization–with all that it implied historically–became anathema, and one author went so far as to write: “We recommend that researchers who use self-report instruments do not use the term “somatisation” (even if the instrument is labeled as a “somatisation” scale), but use the term “multiple physical symptoms” instead. The current operational use may unduly lead to a “psychologisation” of physical complaints” [18].

DSM-5 altered the definitional landscape with the release of the American Psychiatry Association’s highly influential Disease and Statistical Manual-5 (DSM-5) which defined a “mental disease” called *Somatic Symptom Disorder (SSD*) and eliminated somatoform disorders. Briefly, patients had a DSM somatic symptom disorder if they had at least 1 severe somatic symptom (for example, leg or joint pain, headache, etc.) and it was judged that they had at least one of the following: (1) Disproportionate and persistent thoughts about the seriousness of one’s symptoms; (2) Persistently high level of anxiety about health or symptoms; or (3) Excessive time and energy devoted to these symptoms or health concerns. These criteria together with other changes in the DSM unleashed a firestorm of criticism, including the specific concern that the somatic symptom disorder definition would impose a mental illness diagnosis on a large proportion of otherwise normal individuals with physical illnesses [19], [20], [21]. But the key feature of the somatic symptom disorder is not the symptoms–as almost everyone with any severe symptom will qualify; it is that the symptoms are “disproportionate” or “excessive.”

In rheumatic diseases, all patients have symptoms. Patients under the burden of rheumatic illnesses can be overwhelmed and report disproportionate or excessive symptoms. Or they could report severe symptoms that are not disproportionate, or partially disproportionate. Should one believe patients with a “10” score on the 0–10 visual analog pain scale (VAS) when they do not exhibit the behaviors associated with excruciating pain? Does the rheumatoid arthritis patient who doesn’t respond to biologics as she would like have symptom concerns that are excessive? When does the patient’s concern that rheumatoid arthritis is a disabling disorder with increased mortality and disability become excessive? After all, some patients are worried and some are not. Concerns that symptoms are disproportionate or excessive are of particularly of concern for those with fibromyalgia, where there is considerable data and debate among physicians as to whether the symptoms are at least somewhat exaggerated [22], [23], [24], [25]. For patients, a common and important concern is that others think “it is all in my head” [26], [27].

The language of somatization and somatic disorders is confusing. The term *somatization syndrome* disorders has been suggested to identify persons with high levels of somatic symptoms including non-DSM somatoform disorders [28], while DSM-5 somatic symptom disorder implies mental disease and excessive or disproportionate concerns. In this study we describe and evaluate the content and severity of somatic symptoms in patients with rheumatoid arthritis and fibromyalgia. We use the well-regarded Patient Health Questionnaire-15 (PHQ-15) [29] to quantify somatic symptoms and various definitions of somatization syndrome disorders. We explore the DSM-5 criteria and provide some estimates of the proportion of patients who might be considered to have DSM-5 mental illness. Finally we explore the relation of somatic symptoms to fibromyalgia symptoms and diagnosis.

## Methods

### Ethics

All participants were informed about the study procedures and signed an informed consent form. The study was approved by the Via Christi Institutional Review Board of Wichita, Kansas.

### Patients and Diagnosis

In 2012, we administered the PHQ-15 questionnaire to 6, 233 participants with fibromyalgia, rheumatoid arthritis (RA) and osteoarthritis (OA) who were participants in the National Data Bank for Rheumatic Diseases (NDB) longitudinal study of rheumatic diseases outcomes [30]. Participants were volunteers, recruited primarily from the practices of US rheumatologists, who complete mailed or Internet questionnaires at 6-month intervals. They were not compensated for their participation. Diagnoses were made by the patient’s rheumatologist or confirmed by the patient’s physician in cases that were self-referred [30]. However, to be classified as having fibromyalgia patients were required to satisfy research criteria for fibromyalgia by their responses in the most recent survey questionnaire.

Patients were designated as having criteria positive fibromyalgia if they satisfied research criteria for fibromyalgia. [3] The research fibromyalgia criteria (2011) were a modification of the 2010 American College of Rheumatology preliminary diagnostic criteria for fibromyalgia [2] to allow the use of self-report questionnaires for research. When we use the term ACR criteria or (ACR) research criteria for fibromyalgia, we are referring to the modified criteria. For patients to be diagnosed with fibromyalgia they had to have either a Widespread Pain Index(WPI) ≥7 and Symptom Severity Score (SS) ≥5, or a Widespread Pain Index between 3–6 and Symptom Severity Score ≥9. [2], [3] The widespread pain index is a 0–19 count of painful body regions. The Symptom Severity Score is the sum of the *severity* (0–3) of the 3 symptoms (fatigue, waking unrefreshed, cognitive symptoms) plus the sum of the *number* of the following symptoms occurring during the previous 6 months: headaches, abdominal pain, and depression. The final score is between 0 and 12. For fatigue, waking unrefreshed, and cognitive problems, scoring was 0 No problem; 1 Slight or mild problems, generally mild or intermittent; 2 Moderate, considerable problems, often present and/or at a moderate level; 3 Severe: continuous, life-disturbing problems.

Soon after the publication of the 2010 ACR criteria, it was suggested that the 2 components of the 2010 criteria, the 0–19 widespread pain index and the 0–12 symptom severity score, could be combined by addition into a 0–31 index. Originally called the “fibromyalgianess scale” [31], a term that was a little awkward and limiting, it has subsequently been termed the “polysymptomatic distress” scale (PSD). The PSD scale is an excellent measure of the intensity of fibromyalgia symptoms and correlates with all general measures of distress [32]. Because of the scoring rules of the fibromyalgia criteria, criteria positive individuals will always have a PSD score of at least 12. The PSD scale, therefore, provides a way to examine fibromyalgia and fibromyalgia content on a continuous scale.

### Diagnostic Groups

Patients groups with RA (N = 4718) and OA (N = 952) were composed of patients with these diagnoses; they were unselected as to the presence of absence of concomitant fibromyalgia. Patients in the fibromyalgia group (N = 440) were fibromyalgia criteria positive, but did not include patients with RA who satisfied fibromyalgia criteria. Some patients with an initial physician diagnosis of fibromyalgia on entry into the NDB (N = 123) no longer satisfied fibromyalgia criteria at the time of this study, a change that occurred primarily because of symptom improvement [33]. To mimic possible “primary” fibromyalgia as it is seen in the clinic, a noninflammatory group (N = 1515) was composed of patients initially diagnosed with fibromyalgia–whether or not they satisfied criteria for fibromyalgia at any time, as well as patients in the OA group, some of whom might also satisfy fibromyalgia criteria. “OA” was composed of patients with various forms of osteoarthritis and back pain syndromes. OA data are reported in Table 1, primarily to provide perspective for the primary analyses of RA and fibromyalgia that follow.

**Table 1:** Prevalence of PHQ-15 symptoms in RA, SLE and Fibromyalgia.

*PHQ Item*	*RA (4718)*	*OA (952)*	*FM (440)*	*RA (4718)*	*OA (952)*	*FM* * *(440)*
	Bothered a little (%)			Bothered a lot (%)		
Feeling tired or having low energy	75.9	75.2	98.2	29.9	27.6	80.5
Pain in arms, legs or joints	82.3	89.8	98.9	33.1	41.6	73.6
Back pain	66.1	75.5	95.2	24.1	32.3	67.3
Trouble sleeping	61.9	63.6	91.6	21.0	19.5	54.6
Constipation, loose bowels, or diarrhea	44.6	45.2	75.5	9.8	10.9	33.9
Nausea, gas, or indigestion	42.5	43.0	73.2	6.8	7.7	24.1
Headaches	38.4	37.4	73.2	5.5	4.4	22.7
Shortness of breath	29.6	31.3	57.1	4.4	5.1	11.4
Stomach pain	27.7	27.5	60.5	4.3	5.2	16.1
Dizziness	23.8	26.8	57.7	2.6	2.8	7.5
Feeling your heart pound or race	17.5	18.1	41.1	1.6	2.0	6.6
Pain or problems during sexual intercourse	11.8	10.2	22.7	3.9	3.1	10.5
Chest pain	9.5	9.3	28.4	1.1	1.0	3.4
Menstrual cramps or other problems with your periods [Women only]	5.5	4.3	8.6	1.4	1.4	3.0
Fainting spells	1.9	2.5	6.8	0.3	0.6	1.1
Summary Scores						
Total PHQ-15 score Mean (SD)	6.9 (4.5)	7.3 (4.3)	14.0 (4.1)			
PHQ-15 score ≥10 (%)	26.4	28.5	88.9			
≥3 “bothered a lot” symptoms (%)	24.2	26.8	77.5			

PHQ-15– Patient Health Questionnaire-15.

*Criteria positive patients.

### Terminology

We use the recently proposed [28] term “somatization syndromes” to indicate somatization/somatoform disorders when defined by PHQ-15 questionnaire or the PSD scale. DSM-SSD refers to the disorder described by DSM-5. Somatoform disorder is often used in the literature to refer to any somatic symptoms disorder. In this report, somatoform disorder, somatization syndromes, somatic symptom disorders, physical symptoms disorder, bodily distress disorder and functional disorders are terms that have generally similar meanings. When using these terms with the PHQ-15≥10 criterion we do so without implying the presence or absence of psychological reasons for somatic symptoms or a mental disorder, or speculating on whether the symptoms are medically unexplained.

Confusion exists regarding the term “somatic” or “physical” when it is used in the context of somatization syndromes. Each of the syndromes permits consideration of fatigue and sleep problems within its scope of measurement, even if there is uncertainty about whether fatigue and sleep problems are actually “somatic” symptoms. The authors of DSM-5 have also have assessed the DSM-5 somatic symptom disorder diagnosis with the *Somatic Symptom Short Form* that includes fatigue and trouble sleeping [10]. Barsky and Borus indicate that symptoms common to these (functional) somatic syndromes include … problems with memory, attention, and concentration …” [16], thereby including cognitive problems, which are also part of the fibromyalgia criteria [2], [3]. Therefore, in this report we use the term somatic symptoms in the broad sense that includes fatigue, sleep and memory/cognitive problems.

### Study Assessments

#### PHQ-15

We used the Patient Health Questionnaire 15 (PHQ-15) to determine somatic symptom severity and to provide a cut-off for a somatization disorder. The PHQ-15 contains 15 somatic symptoms. Each symptom is scored from 0 (not bothered at all) to 2 (bothered a lot). PHQ-15 scores of 5, 10, and 15 represent cutoff points for low, medium, and high somatic symptom severity, respectively. The usefulness of the PHQ-15 in screening for somatization syndromes and in monitoring somatic symptom severity in clinical practice and research has been demonstrated in numerous studies [29].

A level of PHQ-15≥10 was found to be the optimum level to predict the diagnosis of a “somatoform disorder” in primary care, with a sensitivity of 80.2% and specificity of 58.5% [34]. The PHQ-15 at ≥10 was also used in a large population based study of somatization syndromes where 9.3% were found to satisfy the criterion [28]. In the current report we used this PHQ-15 level to estimate the probable presence of a somatic syndrome disorder. In a similar primary care study of 2,147 eligible patients that utilized the PHQ-15, a cutoff level of 3 or more severe somatic symptoms during the past 4 weeks defined a somatoform disorder with a sensitivity of 78% and specificity of 71% [35]. We also used this cut-off to examine somatic syndrome disorders in our patients.

#### DSM-5 criteria

With respect to the DSM-5 criteria [9], we considered any of the PHQ-15 items that was scored as “bothered a lot” (or “2″) to satisfy the DSM-5 “A” criterion: “ One or more somatic symptoms that are distressing …” As noted above, we also investigated “3 or more severe somatic symptoms” in the PHQ-15 as a more rigorous measure of somatic symptom severity. To investigate the DSM-5 “B” criteria items of “1) Disproportionate and persistent thoughts about the seriousness of one’s symptoms; 2) Persistently high level of anxiety about health or symptoms; and 3) Excessive time and energy devoted to these symptoms or health concerns–we used as a surrogate the reporting of 1, 2 or 3 ACR criteria symptoms that were “3 Severe: continuous, life-disturbing problems.” As there are no precise instructions about how to evaluate the DSM-5 “B” criterion, we also examined mean levels of clinical severity variables described below.

#### Other assessments

Study participants completed the Short-form 36 (SF-36) version 1 from which the physical component summary (PCS) and the mental component summary (MCS) scores were calculated [36], [37]. The primary time period of the SF-36 questionnaire was 4 weeks. The MCS and PCS population mean is 50 with a standard deviation of 10. In addition, using a cutoff score of 42, the MCS had a sensitivity of 74% and a specificity of 81% in detecting patients diagnosed with depressive disorder [38]. For the separate SF-36 5-item mental health scale, the mean score in the lowest tertile in the US population was 61, and was associated in a general increase in health services and mental health specialist care [39]. Patients also self-reported current and lifetime “mental illness” (not defined further in the questionnaire), and the presence now and ever of “depression” and “drug or alcohol abuse.” We classified a patient as having a “psychiatric illness” (current or past) if any “mental illness,” “depression” or “drug or alcohol abuse” was endorsed. These categories were meant to describe self-report patient information about mental health problems, but do not have physician validation.

To measure functional status, we used the Health Assessment Questionnaire disability index (HAQ) [40] and the SF-36 PCS. HAQ scores ≥1 represent substantial functional impairment. Pain over the last week was assessed by a 0–10 visual analog scale. We also used the 5-level Euroqol (EQ-5D) to estimate a preference-based single measure of health status [41]. Lower scores represent worse outcome for the PCS, MCS, and EQ-5D.

Work disability and employment was determined by self-report. We use self-report for disability rather than receipt of a disability pension, as all patients are not eligible for a pension because of age or previous work history limitations. Hospitalization refers to hospitalization for any cause in the previous 6 months and is validated by medical records.

### Statistical Methods

To measure the relation and significance between PHQ-15 groups and predictor variables, we used ordered logistic regression. To measure agreement we used the kappa statistic. According to Landis and Koch [42], kappa may be interpreted as: <0 no agreement, 0.0–0.20 very low agreement, 0.21–0.40 low agreement, 0.41–0.60 moderate agreement, 0.61–0.80 full agreement, and 0.81–1.00 almost perfect agreement.

## Results

Using PHQ-15 scores as the criterion for non-DSM somatic syndrome disorders, 26.4% and 28.5% of RA and OA patients had PHQ-15 scores ≥10 (Table 1). Generally similar values were obtained by using an allied proposed criterion: the percentage of patients who had at least 3 “bothered a lot” endorsements out of 15 PHQ-15 items: 24.2% and 26.8%. When applied to patients with noninflammatory rheumatic disorders, the kappa for agreement between PHQ-15≥10 and “at least 3 bothered a lot” symptoms was 0.762, 95% CI = 0.728–0.795 (complete agreement). The four highest ranked symptoms by occurrence were *feeling tired or having low energy* (fatigue); *pain in arms legs or joints*; *back pain*; and *trouble sleeping*. When “bothered a lot” symptoms in table 1 were studied, PHQ-15 item percentages were approximately three times greater in fibromyalgia than in other diagnoses, providing an estimate of the extent to which patients diagnosed with fibromyalgia differ from those with other rheumatic disorders.

Table 2 demonstrates increasingly abnormal illness status variables with increasing levels of PHQ-15. In this table we restricted analyses to patients with RA, as those patients represent a group selected because they had RA, but unselected with respect to the presence of fibromyalgia or fibromyalgia characteristics. All variable were significantly associated with increasing PHQ-15 categories in ordered logit analyses. As noted above, patients with medium (PHQ-15 = 10–14) and high (PHQ-15≥15) somatic symptom severity satisfied the study somatization syndrome criterion. Younger age patients and women were more likely to be in the more severe categories. Measures of fibromyalgia, including the widespread pain index, the polysymptomatic distress score and fibromyalgia research criteria positivity increased with PHQ-15 somatic symptom severity category. PHQ-15 scores ≥10 showed substantial increases in fibromyalgia prevalence and fibromyalgia-associated scores.

**Table 2:** Characteristics of rheumatoid arthritis patients according to PHQ-15 category.

Variable	PHQ-15 Category			
	PHQ-15 (0–4) Minimal	PHQ-15 (5–9) Low	PHQ-15 (10–14)Medium	PHQ-15 (10–30) High
N (Total = 4718) (%)	1651 (35.0)	1821 (38.6)	928 (19.7)	318 (6.7)
				
Age (years)	65.3 (12.2)	64.0 (12.0)	61.2 (12.8)	57.8 (13.2)
Sex (% male)	26.1	17.9	9.5	9.4
Fibromyalgia survey criteria (+) (%)	1.2	11.3	47.6	84.9
Polysymptomatic distress scale	3.7 (3.5)	8.6 (5.0)	14.2 (5.8)	20.3 (6.4)
Widespread pain index	2.0 (2.7)	4.7 (4.2)	8.0 (5.0)	12.1 (5.3)
PHQ-15	2.4 (1.4)	6.8 (1.4)	11.6 (1.4)	17.0 (2.5)
Mental component score (SF-36)	56.0 (7.6)	50.4 (10.0)	43.8 (11.1)	37.4 (11.3)
Mental health (SF-36)	85.2 (12.5)	76.4 (15.8)	66.4 (18.3)	55.1 (22.5)
“Mental illness” now (%)	0.5	0.9	1.9	6.9
“Mental illness” ever (%)	9.2	8.5	10.9	19.5
Psychiatric disorder now (%)	4.1	12.6	26.6	45.6
Psychiatric disorder ever (%)	30.4	46.8	69.1	81.5
Physical component score (SF-36)	45.3 (9.9)	36.8 (10.2)	30.6 (8.6)	27.9 (7.2)
HAQ (0–3)	0.55 (0.62)	0.97 (0.66)	1.39 (0.61)	1.64 (0.54)
VAS Pain (0–10)	1.8 (1.9)	3.5 (2.4)	5.3 (2.4)	6.4 (2.2)
EuroQol EQ-5D (0–1)	0.84 (0.11)	0.75 (0.12)	0.65 (0.15)	0.55 (0.17)
College graduate (%)	46.5	41.6	34.9	28.6
Current smoker (%)	6.7	8.5	10.8	13.8
Body mass index	26.9 (5.8)	28.7 (7.1)	29.8 (7.7)	31.9 (9.1)
Married (%)	74.2	73.2	69.7	68.2
Employed (%)	34.1	31.3	26.9	24.0
Disabled (%)	5.1	11.6	22.0	35.0
Hospitalized in last 6 months (%)	7.6	10.1	13.9	18.2

PHQ-15– Patient Health Questionnaire-15, HAQ – Health assessment questionnaire.

Continuous scales show mean and (standard deviation). Variables in column 1 predict increases/decreases in severity across PHQ-15 categories at p<0.05.

In addition, self-reported current “mental illness” was approximately 4 times greater in medium PHQ-15 severity and almost 14 times greater compared with the minimal PHQ-15 severity group. Lifetime mental illness varied little across the first 3 categories, but was approximately double in the severe somatic severity group. When mental illness, depression and substance abuse were combined (psychiatric disorder), substantial increases were noted across the PHQ-15 categories. In those with severe somatic symptom reporting, current reported psychiatric illness was 45.6% and lifetime illness was 81.5%. Using Ware’s 42 cut-off for prediction of depressive disorders [38], most such disorders can be seen to occur in those with PHQ-15>10.

PHQ-15 category increases were also associated with substantial increases in pain scores, decreased quality of life and functional ability. These differences are clinically significant and important in the clinical evaluation and care of rheumatoid arthritis. Finally, PHQ-15 increases reflected less education, more cigarette smoking, higher body mass index, more work disability, and an increased rate of hospitalization in the previous six months.

Table 3 approached the relation of somatic symptom severity and clinical variables by classifying RA patients according to the number of symptoms that were “severe, continuous, life-disturbing problems” for the 3 specific symptom variables that are part of the ACR research criteria: fatigue, unrefreshed sleep and cognitive problems. In contrast to the percent of patients in the 4 PHQ categories (35.0%, 38.6%, 19.7%, 6.7%) shown in Table 2, more than 85% of Table 3 patients were in the 0 group. The 4 percentages were 85.2%, 7.1%, 6.3%, and 1.6%. In addition, fibromyalgia criteria positivity was 11.4% in the 0 group, and 56.7%, 75.3%, and 87.1% in the 1, 2 and 3 groups, respectively. Because being >0 indicates a very severe “life-disturbing” symptom, psychological variables were similarly severely abnormal and more so than in the medium and severe categories of PHQ-15 shown in Table 2. Overall, the major change in severity variables in Table 3 occurs between the 0 and 1 group. For most variables in Table 3, categories of 1, 2 and 3 are associated with small, but progressively more abnormal symptoms and characteristics.

**Table 3:** Characteristics of patients with rheumatoid arthritis according the number of severe; continuous, life-disturbing problems with fatigue, unrefreshed sleep or cognition.

*Variable*	*Count*			
Number of severe; continuous, life-disturbing problems forFatigue, Unrefreshed sleep or cognitive problems	0	1	2	3
N (Total = 4780) (%)	4071 (85.2%)	338 (7.1%)	301 (6.3%)	70 (1.6%)
Age (years)	64.4 (12.3)	60.6 (12.7)	58.0 (12.9)	55.7 (11.8)
Sex (% male)	20.2	11.2	7.6	5.7
FM research criteria (%)	11.4	56.7	75.3	87.1
Polysymptomatic distress scale	7.3 (5.8)	15.3 (5.9)	18.1 (6.4)	21.9 (6.7)
Widespread pain index	4.2 (4.4)	8.0 (5.3)	9.2 (5.9)	11.2 (6.3)
PHQ-15	6.0 (3.9)	10.9 (4.1)	12.4 (4.4)	14.2 (5.4)
Mental component score (SF-36)	52.0 (9.9)	41.7 (11.0)	38.9 (11.7)	34.4 (11.7)
Mental health 5-item (SF-36)	78.8 (16.0)	63.7 (19.7)	59.0 (21.6)	49.9 (24.8)
“Mental illness” now (%)	0.8	3.0	5.0	11.4
“Mental illness” ever (%)	8.8	14.5	16.6	24.3
Psychiatric disorder now (%)	10.5	33.1	38.2	60.0
Psychiatric disorder ever (%)	42.8	71.9	77.4	90.0
Physical component score (SF-36)	39.6 (11.0)	29.8 (8.6)	27.0 (7.9)	28.0 (6.8)
HAQ (0–3)	0.84 (0.69)	1.48 (0.59)	1.66 (0.57)	1.79 (0.60)
VAS Pain (0–10)	3.0 (2.4)	5.6 (2.4)	6.5 (2.2)	7.1 (2.2)
EuroQol EQ-5D (0–1)	0.78 (0.12)	0.62 (0.16)	0.55 (0.17)	0.48 (0.19)
College graduate (%)	42.6	34.0	28.6	30.0
Current smoker (%)	7.7	12.4	15.3	21.4
Body mass index	28.0 (6.8)	30.5 (7.4)	31.4 (8.8)	31.5 (8.8)
Married (%)	73.9	63.0	67.4	57.1
Employed (%)	31.7	26.4	24.9	20.3
Disabled ((%)	9.2	29.7	36.9	43.5
Hospitalized in last 6 months (%)	9.5	14.8	16.3	18.6

PHQ-15– Patient Health Questionnaire-15, HAQ – Health assessment questionnaire.

Continuous scales show mean and (standard deviation).

Table 4 applies the 0–3 count test of Table 3 to noninflammatory patients who satisfy fibromyalgia criteria, with the goal of trying understand if applying the count variables provides additional information about somatic symptoms and psychological variables, as well as with ordinary clinical variables. The data tend to suggest that once the fibromyalgia criteria are satisfied, the count of very severe symptoms provide only a relatively equal small to moderate step-wise increase in variable severity. Of interest, the increase across the 0–3 categories is associated with small increases in the PHQ-15 of 1.1, 0.9, 1.2; and of 2.1, 1.1 and 1.3 in the longer (0–31) PSD scale.

**Table 4:** Characteristics of 440 patients with criteria positive ‘primary’ fibromyalgia according the number of severe; continuous, life-disturbing problems with fatigue, unrefreshed sleep or cognition.

*Variable*	*Count*			
Number of severe; continuous, life-disturbing problems forFatigue, Unrefreshed sleep or cognitive problems	0	1	2	3
N (Total = 440) (%)	214 (48.6%)	92 (20.9%)	88 (20.0%)	46 (10.5%)
Age (years)*	62.8 (11.8)	61.0 (12.6)	56.5 (12.5)	53.3 (11.2)
Sex (% male)	11.7	9.8	9.1	6.5
FM research criteria (%)	100.0	100.0	100.0	100.0
Polysymptomatic distress scale*	18.1 (4.3)	20.2 (4.6)	21.3 (5.1)	22.6 (6.1)
Widespread pain index	11.6 (4.0)	12.4 (4.2)	11.8 (4.8)	11.8 (5.7)
PHQ-15*	12.1 (3.8)	13.2 (3.7)	14.1 (3.9)	15.3 (5.6)
	(64.0%)	61.4%	55.4%	52.2%
Mental component score (SF-36)*	42.9 (11.0)	40.9 (11.1)	37.3 (11.5)	31.3 (13.2)
Mental health (SF-36)*	65.6 (18.4)	61.8 (20.0)	57.2 (20.8)	42.7 (25.8)
“Mental illness” now (%)*	2.8	6.5	8.0	23.9
“Mental illness” ever (%)	17.8	20.7	19.3	34.8
Psychiatric disorder now (%)*	38.8	35.9	35.2	60.9
Psychiatric disorder ever (%)	77.1	82.6	81.8	89.1
Physical component score (SF-36)*	30.3 (8.5)	28.3 (8.7)	27.9 (6.9)	28.1 (9.1)
HAQ (0–3)*	1.25 (0.61)	1.47 (0.59)	1.35 (0.59)	1.54 (0.67)
Pain (0–10)*	5.8 (2.0)	6.7 (2.2)	7.0 (1.9)	7.3 (2.2)
EQ-5D*	0.65 (0.15)	0.57 (0.17)	0.57 (0.16)	0.47 (0.20)
College graduate (%)	35.0	34.8	36.4	28.3
Current smoker (%)	12.1	15.2	17.0	17.4
Body mass index	31.2 (7.4)	32.0 (9.2)	32.0 (7.4)	30.4 (8.7)
Married (%)	71.5	64.1	67.0	65.2
Employed (%)*	22.4	25.0	34.1	26.1
Disabled (self-reported work status) (%)*	27.1	31.5	23.9	34.8
Hospitalized (%)	11.7	15.2	19.3	13.0

PHQ-15– Patient Health Questionnaire-15, HAQ – Health assessment questionnaire.

Continuous scales show mean and (standard deviation).

* = p<0.05.

### The Relation of PHQ-15 to PSD, and the Concept of Fibromyalgia as a Somatic Symptom Disorder

**Figure 1:**
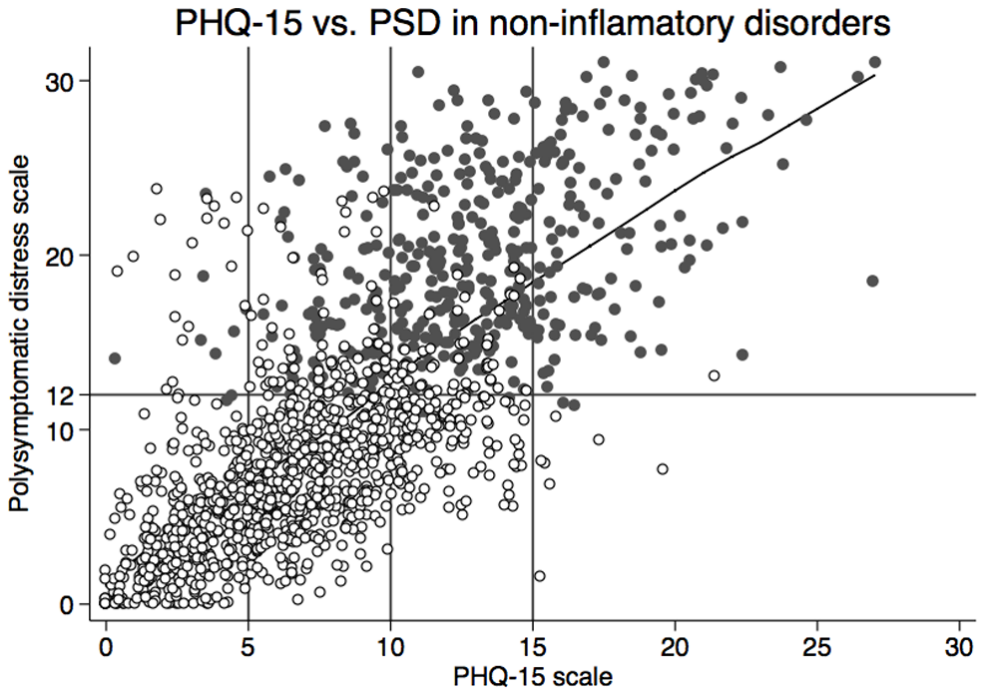
The relation of the PHQ-15 and Polysymptomatic Distress (PSD) scales. The vertical lines at 5, 10 and 15 define the PHQ-15 severity groups (0–4, 5–9, 10–14, ≥15). The horizontal line at 12 roughly separates fibromyalgia negative patients (<12) from mostly fibromyalgia criteria positive patients (≥12). A small amount if random jitter is added to the symbols to make overlapping symbols easier to see.

When examined across the full length of the PHQ-15 and PSD scales in the RA patients of Table 2 and 3, as well as in patients with non-inflammatory disorders, the Pearson correlation coefficient between PHQ-15 and PSD was 0.74. We also evaluated the ability of the PSD and PHQ-15 scales to predict criteria positive fibromyalgia. The area under the receiver operating curve (AUC-ROC) was 0.97 for PSD and 0.89 for PHQ-15. Using a cutpoint of 10 on the PHQ-15 scale, the sensitivity and specificity of PHQ-15 for fibromyalgia diagnosis was 80.9% and 80.0% (correctly classified = 80.3%) compared with 84.3% and 93.7% (correctly classified = 91.7%) for the PSD scale. The kappa value for agreement in diagnosis was 0.57 (moderate agreement). The relation of PHQ-15 to PSD can be seen graphically in Figure 1. The vertical lines at 5, 10 and 15 show the PHQ-15 groups. The horizontal line at 12 roughly separates fibromyalgia negative patients (<12) from mostly fibromyalgia criteria positive patients (≥12). Patients with fibromyalgia whose PHQ-15 score is <10 (PHQ-15 0–9, PDS ≥12) are misclassified by PHQ-15 because their symptom score is too low; they satisfy ACR research criteria by virtue of a high widespread pain index. In addition, patients with PHQ-15≥10 who do not meet ACR criteria fail because they have too low of a WPI score. These data show that PHQ-15 and PSD are similar in their ability to identify the same type of patients, differing only in the number of painful sites that are required by the ACR research criteria.

## Discussion

The data of this study demonstrate the extent to which fibromyalgia can be labeled a somatic symptom disorder or what Kroenke has labeled a “physical symptom disorder:” Such a disorder “would consist of one or more physical symptoms currently present, not fully explainable by another medical or psychiatric disorder (with the exception of functional somatic syndromes), causing functional impairment. Duration must be at least 6 months, and severity could be graded as mild, moderate, or severe using a 15-symptom checklist (PHQ-15). Finally, the type of symptoms or symptom syndromes present in the patient could be specified. Conclusions: PSD should be considered as a simpler and more inclusive diagnosis to replace several somatoform diagnoses currently in use” [15]. When characterized that way 89.9% of those with fibromyalgia have PHQ-15 scores ≥10, the level others have suggested is a requirement for somatic symptom syndromes [28], [34]. In addition, as shown in Table 2, patients with this disorder have substantially abnormal scores for functional status and physical and mental health.

In a previous population based study, we have shown that fibromyalgia can be considered to be a dimensional or continuum disorder rather than a discrete illness, and that the polysymptomatic distress (or fibromyalgianess) scale provides a general, useful measure of the fibromyalgia continuum [32]. The PHQ-15 and the polysymptomatic distress scale tap into the same somatic dimension, and are correlated at 0.74. The essential difference between the scales is that the polysymptomatic distress scale weights pain, as measured by the widespread pain index, more than the PHQ-15 does. In noninflammatory patients satisfying the fibromyalgia criteria, joint and back pain account for a 26% of the total PHQ-15 score, while the WPI accounts for 58% of the polysymptomatic distress score. These differences can be appreciated in the agreements/non-agreements of Figure 1. Overall, these findings fit with the idea that fibromyalgia is a pain-predominant somatic symptom disorder.

Much current thought suggests that, as Wessely put it, “There is only one functional somatic syndrome” [43]. “… a substantial overlap exists between the individual syndromes and that the similarities between them outweigh the differences” “…existing definitions of these syndromes in terms of specific symptoms is of limited value; instead we believe a dimensional classification is likely to be more productive” [44]. This view, nuanced differently by different authors, is reflected in much current thinking [5], [15], [16], [17], [45], [46]. In addition, the current predominant emphasis on pain extent (severity) in fibromyalgia was new to fibromyalgia/fibrositis, beginning in the 1980s. In fact, the dominant Yunus criteria that preceded the 1990 ACR criteria emphasized symptoms over pain [27], [47]. These observations, with respect to the dimensional nature of fibromyalgia-type symptom severity and the dimensional nature of pain/symptom inclusion suggest that fibromyalgia is a more mutable, uncertain concept than often acknowledged. That is, the content of fibromyalgia is somewhat variable and is dimensional. Like the PHQ-15 at low levels of severity (PHQ-15 = 5–9), the polysymptomatic distress scale tracks symptoms to below syndromal levels. We believe that neuroscience investigators who rely on fibromyalgia as a hard diagnosis with specific cut-off, may be tapping instead into a common core of functional syndromes that is essentially dimensional. It may well be that the investigation of neuroscience issues would more appropriately be directed over the range of the polysymptomatic distress and PHQ-15 scales.

The DSM-5 somatic symptom disorder differs in at least one important way from the syndromes described above: it perforce describes a mental disease. Given one severe, persistent somatic symptom, the diagnosis of DSM-5 SSD in patients with rheumatic diseases depends, in all practical cases, in satisfying the mandate of at least one of the following: (1) Disproportionate and persistent thoughts about the seriousness of one’s symptoms; (2) Persistently high level of anxiety about health or symptoms; and (3) “Excessive time and energy devoted to these symptoms or health concerns. “Disproportionate,” “high levels” and “excessive time” are judgments. They can be determined, according to the DSM, by “clinical experience, training and judgment based on guidance such as that contained in the DSM-5 text to recognize when a patient’s thoughts, feelings and behaviors are indicative of a mental disorder” [14]. To test this judgment, the APA committee carried on clinical trials at a single psychiatric clinic site using approximately 420 psychiatric patients who were currently symptomatic for any DSM-IV diagnoses or high-probability symptoms associated with the DSM-5 diagnoses. Forty-two subjects had an SSD-like disorder (Complex somatic symptom disorder) [48], [49]. Although the “SSD was found to have very good reliability [10], only previously diagnosed psychiatric patients were studied, no patients had disorders like rheumatoid arthritis or fibromyalgia (to our understanding), and physician examiners were single-center professors and allied psychiatric staff. From this we conclude that the ability to reliably and validly identify SSD patients in rheumatic disorders, including rheumatoid arthritis and fibromyalgia, was not tested; and that available reliability data cannot be extrapolated to such patients.

The data of the current study showed that psychological and psychiatric problems increased with increasing PHQ-15 and polysymptomatic distress scores. We also found that 51.4% of patients with fibromyalgia and 14.8% with RA had fatigue, sleep or cognitive problems that were severe, continuous, and life-disturbing (Tables 3 and 4). Among those with fibromyalgia (Table 4) there was evidence of self-report of severe functional, pain and quality of life disturbances. Whether these findings represent “disproportionate,” “high levels” or “excessive time” could be a matter of conjecture, but a strong case can be made that these data suggest that almost all patients with fibromyalgia meet the DSM-5 B criterion, as do a substantial fraction of those with RA.

There are additional reasons to believe that validity and reliability of the DSM SSD may be unsatisfactory in persons with illnesses like fibromyalgia and rheumatoid arthritis. Research and clinical data are split on whether fibromyalgia patients exaggerate [22], [23], [24], [25], including whether rheumatoid arthritis patients with fibromyalgia “over-report.” A study of the RA Disease Activity Index concluded that “The DAS28 [50] [a measure of disease activity in RA], as expected, proved to be inappropriate to express disease activity in FM patients. DAS28 values for expressing disease activity in RA patients may be flawed by coexisting FM …” [51]. Patients indicate that unsympathetic and non-understanding physicians are an important problem for them [52]. It is likely that some assessors would rate rheumatic patients as disproportionate with regard to intensity of symptoms while others would find patients adequately model their predicament. In addition, there is no clear definition of a “serious” illness. So it is possible that the presence of a mental illness may depend more on the examiner than the patient. The 14-item Whitely index [53] has been used (and recommended by DSM authors) as a method to assess disproportionate and excessive features. But it contains questions like “Do you often worry about the possibility that you have got a serious illness? Are you bothered by many aches and pains? Do you find that you are often aware of various things happening in your body? Is it hard for you to believe the doctor when he tells you there is nothing for you to worry about? Do you get the feeling that people are not taking your illness seriously enough?” The questions appear to lack face validity when assessing symptoms in fibromyalgia (and other rheumatic illnesses), and the expected answers should be “yes” Overall, our data suggest that fibromyalgia meets non-DSM criteria for somatization syndromes, and those with fibromyalgia report severe physical and psychological symptoms. But we are dubious that the DSM-5 approach can distinguish validly and reliably which fibromyalgia patients are and which are not mentally ill, particularly in clinical care settings where diagnosis will come most often from generalists.

## Supporting Information

Table S1 **DSM-5 Somatic Symptom Disorder (SSD).**
(DOCX)
