# Supplementary material for: Symptoms, the Nature of Fibromyalgia, and Diagnostic and Statistical Manual 5 (DSM-5) Defined Mental Illness in Patients with Rheumatoid Arthritis and Fibromyalgia
Source: PLoS One. 2014 Feb 14;9(2):e88740. doi: 10.1371/journal.pone.0088740 (PMC3925165; doi:10.1371/journal.pone.0088740)
Supplement: Table S1 — DSM-5 Somatic Symptom Disorder (SSD). (DOCX) [file pone.0088740.s001.docx]

**Table S1. DSM-5 Somatic Symptom Disorder (SSD)**

| ***A***. One or more somatic symptoms that are distressing and/or result in significant disruption of daily life. |
| --- |
| ***B.*** Excessive thoughts, feelings, or behaviors related to the somatic symptoms or associated health concerns as manifested by at least one of the following:   (1) Disproportionate and persistent thoughts about the seriousness of one’s symptoms.  (2) Persistently high level of anxiety about health or symptoms  (3) Excessive time and energy devoted to these symptoms or health concerns |
| ***C***. Although any one symptom may not be continuously present, the state of being symptomatic is persistent (typically more than 6 months). |

Specify if:

***With predominant pain*** (previously pain disorder). This specifier is for individuals whose somatic symptoms predominantly involve pain.

Specify if:

***Persistent***: A persistent course is characterized by severe symptoms, marked impairment, and long duration (more than 6 months).

Specify current severity:

***Mild***: Only one of the symptoms specified in Criterion B is fulfilled.

***Moderate***: Two or more of the symptoms specified in Criterion B are fulfilled.

***Severe***: Two or more of the symptoms specified in Criterion B are fulfilled, plus there are multiple somatic complaints (or one very severe somatic symptom).
